# Risk reduction of cognitive decline and dementia: Update of World Health Organization (WHO) guidelines for Hormone Replacement Therapy (HRT) and Traumatic Brain Injury (TBI)

**DOI:** 10.1002/alz70861_107921

**Published:** 2025-12-23

**Authors:** Chris Fox, Aimee Spector, Georgina Charlesworth, Martha Hickey, Huw Williams, Roopal Desai, Dafinah Azman, Melissa Melville, Hope Kent, Nicholas Smith, Lexi He, Katrin M. Seeher, Primrose Nyamayaro

**Affiliations:** ^1^ University of Exeter, Exeter, Devon UK; ^2^ NIHR Healthtech research centre, Exeter UK; ^3^ University College London, London, England UK; ^4^ University College London, London UK; ^5^ University of Melbourne, Melbourne Australia; ^6^ UCL, London UK; ^7^ North East London Foundation NHS Trust, London, Ilford UK; ^8^ University of Exeter, Exeter UK; ^9^ World Health Organization, Geneva Switzerland; ^10^ UNSW Sydney, Sydney Australia; ^11^ Global Brain Health Institute Trinity College Dublin, Dublin Ireland; ^12^ WHO, Geneva Switzerland

## Abstract

**Background:**

There is clear evidence for key dementia risk factors which impact on trajectory with 45% of cases attributable to modifiable risk (Livingston 2024). The WHO has commissioned a series of state of the art systematic reviews (*n* =16) on dementia risk reduction to update 2019 recommendations. This session presents WHO state of the art reviews on hormone replacement therapy (HRT) and interventions for Traumatic Brain Injury (TBI) in cognitive decline and dementia.

**Review Questions**:

In the menopause transition, cognitive impairment is a common (prevalence 30‐66%). However, there is contradictory evidence on the relationship between Hormone Replacement Therapy (HRT) and dementia. This review therefore answers the question:

*For woman with Premature Ovarian Insufficiency or in early, peri or post‐menopause (with normal cognition or mild cognitive impairment); does current or previous HRT use compared to no HRT impact upon the risk of cognitive decline and/or dementia?Prospero‐CRD42025639384*

Several meta‐analyses of population‐level cohort studies have demonstrated an increase in dementia risk following TBI (Gardner et al., 2022;Gu et al., 2023). However, the efficacy of pharmacological and non‐pharmacological interventions post‐TBI to reduce this risk is unclear. The TBI review therefore answers the question:

*What interventions aimed at prevention/treatment of TBI (e.g. Policies, legislation, concussion protocols, neuro‐rehabilitation and pharmacological treatment) are effective in reducing the risk of subsequent cognitive decline and/or dementia?Prospero‐CRD420251027658*

**Method:**

Systematic review and meta‐analysis. Search strategies implemented across Embase, Cochrane, Medline and PsycInfo, using the relevant subject headings.

**Results:**

**TBI review**: 13883 papers identified and after duplicates 8699 were screened, of which 425 were considered at the full text screening stage and 30 were considered for analysis.

**HRT review**: 5381 papers were identified and after duplicates 3150 were screened, of which 162 were considered at the full text screening stage and 13 were considered for analysis.

Key findings will be presented.

**Conclusion:**

In 2024 the lancet commission (Livingston 2024) reported the evidence was unclear whether menopause and HRT are causally related to dementia risk and suggested that TBI increases dementia risk. In this presentation we report the latest findings on the role of HRT in cognitive decline/dementia and the role of preventative interventions in those with TBI.

